# Data for ampholytic ion-exchange materials coated with small zwitterions for high-efficacy purification of ionizable soluble biomacromolecules

**DOI:** 10.1016/j.dib.2018.09.102

**Published:** 2018-10-03

**Authors:** Jingjing Rao, Juan Liao, Youquan Bu, Yitao Wang, Xiaolei Hu, Gaobo Long, Mingtong Huang, Luhui Zhong, Xiaolan Yang, Fei Liao

**Affiliations:** a College of Laboratory Medicine, Chongqing Medical University, Chongqing 400016, China; b Yongchuan Hospital, Chongqing Medical University, Chongqing 402160, China; c College of Basic Medicine, Chongqing Medical University, Chongqing 400016, China; d Chongqing Bolanying Biotechnology Co. Ltd., Xiyong, Shapingba, Chongqing 401332, China; e School of Pharmacy and Bioengineering, Chongqing University of Technology, Chongqing 400054, China

## Abstract

Data in this article are associated with the research article “Ampholytic ion-exchange materials coated with small zwitterion for high-efficacy purification of ionizable soluble biomacromolecules” (Rao et al., 2018) [1]. This article provided data on how to design ampholytic ion-exchange material (AIEM) for the purification of ionizable soluble biomacromolecules for both high activity yields and favorable homogeneity, with two uricases as protein models and a plasmid as DNA model. Data were made publicly available for further analyses.

**Specifications table**

**Table:** 

Subject area	*Chemistry.*
More specific subject area	*materials for biomolecule engineering.*
Type of data	*Table, text file, graph, Figure.*
How data was acquired	*Biotek ELX 800 and BIOTEK EON microplate readers and Nanodrop 1000 to record the adsorption, Biorad CFX96 for qPCR process curves.*
Data format	*Raw, analyzed.*
Experimental factors	*For adsorption of plasmid and the targeted protein, lysates were prepared in an indicated adsorption buffer by sonication treatment for 5.0 min at 0* °C, *which after further centrifugation served as the samples.*
Experimental features	*Three AIEMs with optimized ratios of ampholytic groups were prepared from magnetic solid supports of Chongqing Bolanying Biotechnology Co. Ltd, for adsorption of uricases or its plasmid via electrostatic attraction and elution via electrostatic repulsion, the isolation efficiency was compared with other commercial ion-exchange media.*
Data source location	*Chongqing Medical University, Chongqing 400016, China*
Data accessibility	*Data are available with this article.*

**Value of the data**

• Supporting the high efficacy of AIEM for the purification of uricases via the reversal of the types of net charges of uricases to realize electrostatic repulsions for elution.
• Supporting the high efficiency of AIEM for solid phase extraction of plasmid via the reversal of the types of net charges of AIEM to fulfil electrostatic repulsions for elution.
• Supporting the incomparable advantages of AIEM for the purification of applicable ionizable soluble biomacromolecules over other classical ion-exchangers.

## 1 Data

**Fig. 1:**
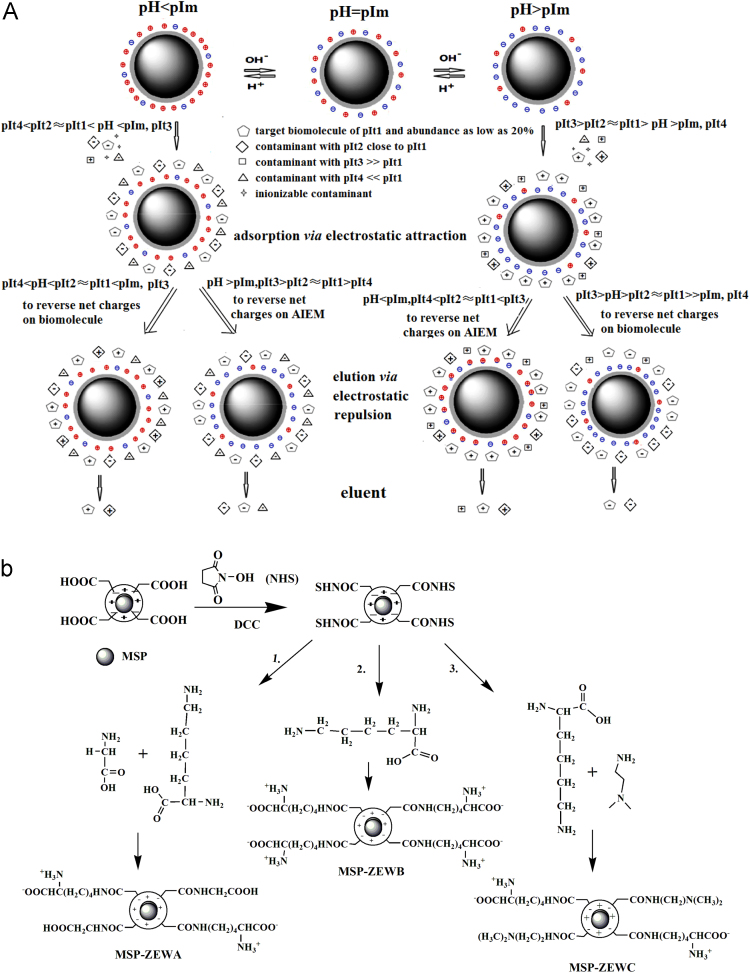
Three types of net charges on the designed AIEMs and their associations with the elution of the adsorbed biomacromolecules (a) and preparation route of the designed AIEMs bearing dynamic pIms (b).

**Fig. 2:**
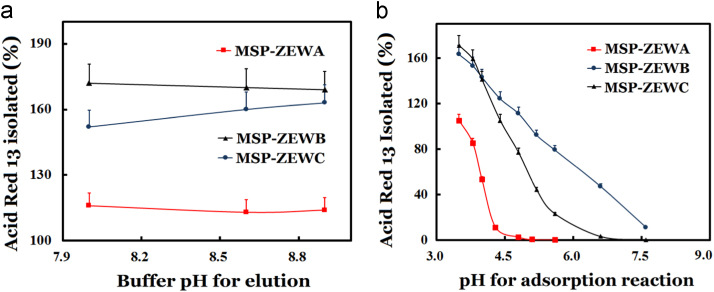
Effects of pH on the adsorption and elution of acid red 13.The adsorptions with AIEMs in 5.0 min used 0.80 ml of 0.20 M sodium acetate at an indicated pH while the elution in 5.0 min utilized 0.80 ml of 20 mM Tris–HCl at an indicated pH. The absorbance of the probe was measured at 506 nm with a spectrophotometer. Error bars were given for assays in triplicate, which showed the coefficients of variations usually below 5%.

**Fig. 3:**
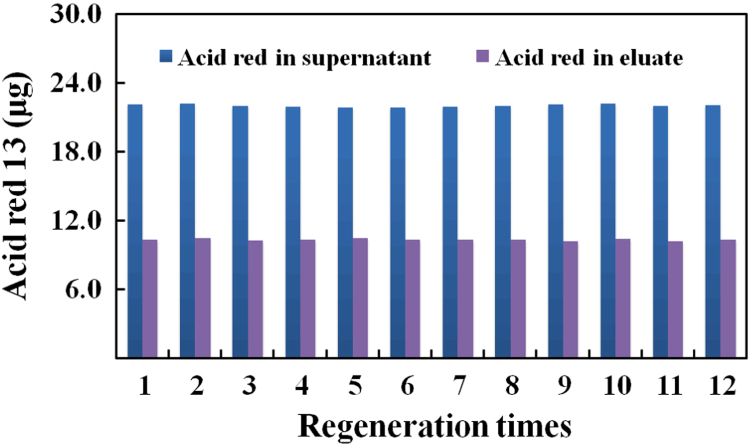
Adsorptions of acid red 13 on two batches of MSP-ZEWB after 12 recycling uses.The adsorptions with AIEMs in 5.0 min used 0.80 ml of 0.20 M sodium acetate at pH 3.6 while the elution in 5.0 min utilized 0.80 ml of 20 mM Tris–HCl at pH 8.9. The absorbance was measured at 506 nm.

The data in this article provides information on how to design the ampholytic ion-exchange materials (AIEM) coated with small zwitterions and ampholytic groups (Fig. 1). The data also showed that AIEMs had different pIms (Fig. 2), the unchanged isolation capacity of ions as indexed by acid red 13 after 12 recycling uses and regeneration of AIEM (Fig. 3).

**Fig. 4:**
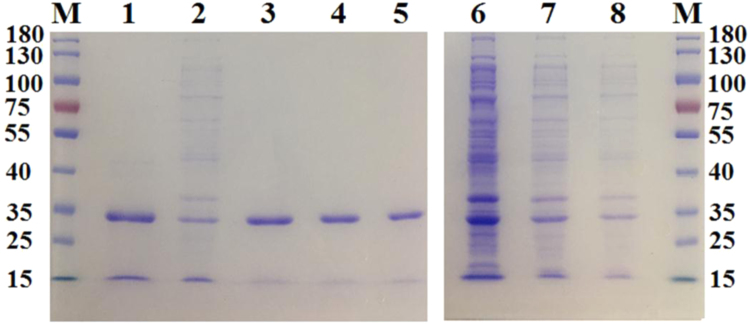
Examination of homogeneity of MGU in eluents from MSP-ZEWB. M: Molecular weight Marker of proteins; 1: Wildtype MGU purified carefully following those described in [2], [3], ~ 5 μg; 2: Cell lysates of crude MGU, ~5 μg; 3: The 3rd eluent with 0.20 ml lysate, ~ 5 μg; 4: The 3rd eluent with 1.0 ml lysate, ~ 5 μg; 5: The 3rd eluent with 4.0 ml lysate, ~ 5 μg; 6: Cell lysate of crude MGU, ~ 45 μg of total proteins; 7: Cell lysate of crude MGU, ~ 15 μg of total proteins; 8: Cell lysate of crude MGU, ~5 μg of total proteins.

**Fig. 5:**
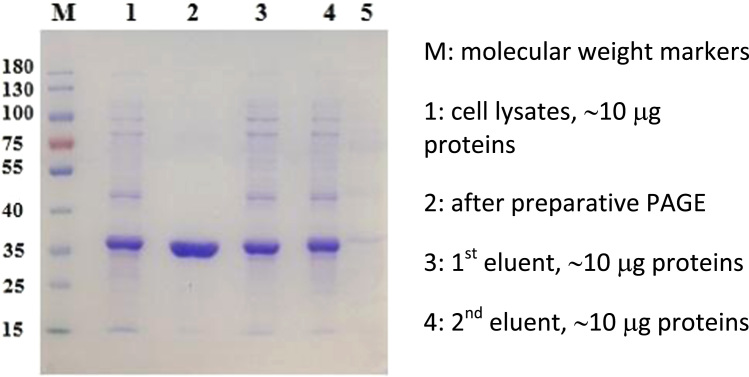
SDS-PAGE analysis of BFU after purification with 5.0 mg MSP-ZEWC.See Ref. [4] for the purification of BFU.

The data were provided to show the performance of the designed AIEMs for purification of uricases via the reversal of the type of net charges of uricases to change from electrostatic attractions for adsorptions to electrostatic repulsions for elution (Table 1, Table 2, Figs. 4 and 5), and the performance of AIEMs for the purification of a plasmid via the reversal of the type of net charges of the AIEM to fulfil electrostatic repulsions for elution (Table 4, Table 5, Fig. 7, Fig. 8).

**Table 1:** Elution of MGU at pH 10.0 in batch mode after adsorption at pH 7.8 on 10.0 mg MSP-ZEWB.

Sample quantity	0.20 ml of cell lysate		1.0 ml of cell lysate			4.0 ml of cell lysate	
Purification steps	activity(kU/L)	protein(g/L)	activty(kU/L)		protein(g/L)	activity(kU/L)	protein(g/L)
Cell lysate	3.7	3.5	3.7		3.5	3.7	3.5
After adsorption	2.5	2.7	2.9		3.2	3.4	3.3
1^st^ eluent, 0.40 ml	0.16	~ 0.02	0.16		0.07	0.24	0.07
2^nd^ eluent, 0.10 ml	0.24	0.09	0.57		0.08	0.53	0.07
3^rd^ eluent, 0.20 ml	0.30	~ 0.027	0.39		0.039	0.28	0.046
4^th^ eluent, 0.20 ml	0.05	~ 0.02	0.08		~ 0.01	0.09	~0.01
Eluted (U or mg)	0.16	0.024	0.22	0.044		0.22	0.045
Total yield (%)	66	15	29	15		18	6
Highest activity (kU/g)	11.0 ± 2.5 (*n* = 2)		10.0 ± 2.2 (*n* = 2)			8.9 ± 2.4 (*n* = 2)	
Highest purification	11.0 ± 2.3 (*n* = 2)		9.0 ± 2.4 (*n* = 2)			8.1 ± 2.8 (*n* = 2)	

The parameter for that after adsorption was the catalytic activity or protein concentration in the supernatant after the separation of MSP. Total activity yield was the percentage of the eluted activity to that adsorbed on MSP-ZEWB. Proteins in the 1^st^ eluents were quantified by absorbance at 280 nm with Nanodrop 1000, while those in the other eluents were determined after concentration to just 20 μl *via* lyophilization.

**Table 2:** Elution of BFU at pH 9.2 in Batch mode after adsorption at pH 6.3 on 10.0 mg MSP-ZEWC.

Sample quantity	0.10 ml of cell lysate		2.0 ml of cell lysate	
Purification steps	Activity (kU/L)	Protein (g/L)	Activity (kU/L)	Protein (g/L)
Cell lysate	0.37	3.4	0.52	4.0
After adsorption	Undetectable (UD)	UD	0.42	3.5
1^st^ eluent, 0.20 ml	0.10	0.89	UD	UD
2^nd^ eluent, 0.10 ml	0.05	0.49	UD	UD
Eluted (U or mg)	0.025	0.22	UD	
Total yield (%)	67	68	/	
Highest activity (kU/g)	0.11		/	
Highest purification	1.2 ± 0.2 (*n* = 2)		/

**Fig. 6:**
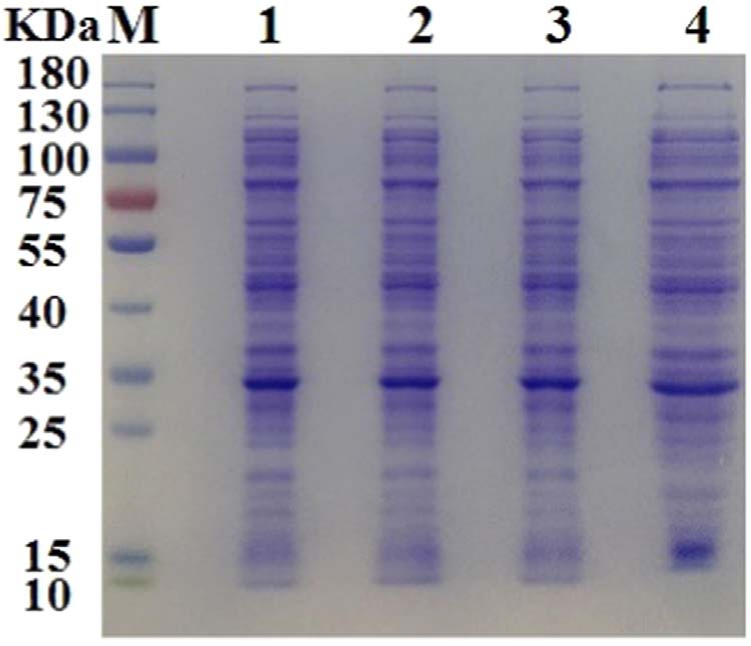
SDS-PAGE analysis of MGU after purification with 0.20 ml Toyopearl SP-650C M: molecular weight marker; 1: the first eluent with 0.20 ml cell lysates; 2: the first eluent with 1.0 ml cell lysates; 3: the first eluent with 5.0 ml cell lysates. In each lane of the loaded sample, the total activity of MGU was the same so that MGU band density was consistent.

**Fig. 7:**
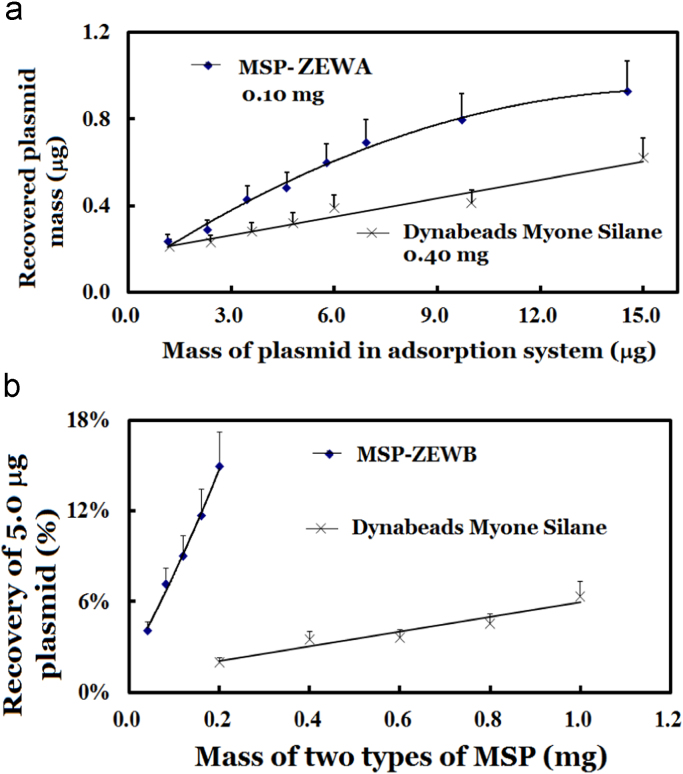
Comparison of plasmid isolation with the designed AIEMs and silane-functionalized MSP.The adsorption of plasmids employed 0.20 ml of 0.20 M sodium acetate at pH 3.6 and the elution utilized 50 μl of 20 mM Tris–HCl buffer at pH 8.9. The plasmid was quantified with Nanodrop 1000 by absorbance at 260 nm. Error bars were given for assays in duplicate, which showed the coefficients of variations usually close to 15%.

**Fig. 8:**
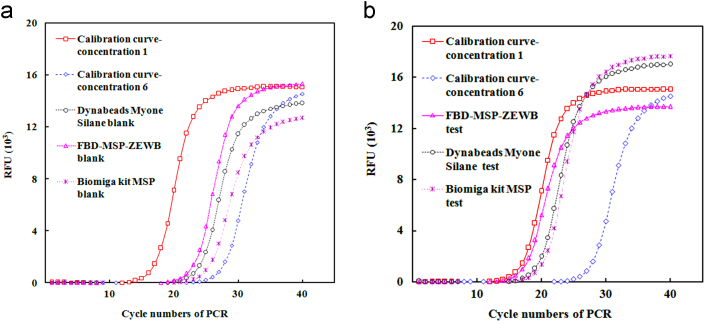
qPCR analysis of the plasmid extracted with different MSPs. (See Ref. [1] for details).

Data were provided to show the advantages of AIEMs over classical ion-exchangers for the purification of uricases (Table 3 and Fig. 6) and of the plasmid (Table 4, Table 5, Figs. 7 and 8).

**Table 3:** Elution of MGU at pH 10.0 in batch mode after adsorption at pH 7.8 on 0.20 ml stacked gel of Toyopearl SP-650C.

Sample quantity	0.20 ml crude sample		1.0 ml crude sample			5.00 ml crude sample		
Purification steps	Activity(kU/L)	Protein(g/L)	Activty(kU/L)		Protein(g/L)	Activity(kU/L)		Protein(g/L)
Cell lysate	3.1	4.3	3.1		4.3	3.1		4.3
After adsorption	2.1	2.7	2.6		3.5	2.7		3.9
1^st^ eluent, 0.40 ml	0.45	0.67	0.56		0.70	0.70		0.80
2^nd^ eluent, 0.20 ml	0.16	0.26	0.18		0.33	0.25		0.34
3^rd^ eluent, 0.10 ml	0.08	ND	0.09		ND	0.13		ND
Eluted (U or mg)	0.21	0.32	0.27	0.35		0.34	0.38	
Total yield (%)	~ 100	97	54	44		11	19	
Highest activity (kU/g)	0.6		0.8			0.9		
Highest purification	no		1.1 ± 0.2 (*n* = 2)			1.2 ± 0.2 (*n* = 2)		

The parameter for after adsorption was the catalytic activity or protein concentration in the supernatant after the separation of MSP. Total activity yield was the percentage of the eluted activity to that adsorbed on MSP-ZEWB. Proteins in the 1^st^ eluents were quantified by absorbance at 280 nm with Nanodrop 1000, while those in the other eluents were determined after concentration to just 20 μl *via* lyophilization.

**Table 4:** qPCR comparison of the purified plasmid of MGU isolated with MSP (*n* = 2).

Sample	Dynabeads Myone Silane blank	MSP-ZEWB blank	Biomiga kit MSP blank	Dynabeads Myone Silane test	MSP-ZEWB test	Biomiga kit MSP test
Ct	25.56 ± 0.23	24.45 ± 0.31	25.74 ± 0.25	20.46 ± 0.33	18.60 ± 0.21	21.01 ± 0.41
Plasmid (pg)	0.43	0.94	0.35	14.09	51.88	9.58
Ratio	1.0	2.2	0.81	1.0	3.7	0.68

Response curve/calibration curve with the reference of the plasmid						
Plasmid (pg)	60	15	3.75	0.94	0.24	0.06
Ct	18.40 ± 0.16	20.36 ± 0.25	22.31 ± 0.15	24.23 ± 0.22	26.57±0.35	28.08 ± 0.25
Equation for the response curve			Ct = 24.2337 − 1.406625 × ln(m) *R*^2^ > 0.998			

Data in duplicate were the same as shown in Fig. 8.

**Table 5:** Recovery of bases, nucleosides and nucleotides with different separation media.

Compounds/conditions	Dynabeads Myone Silane, 1.0 mg each time		Spin silane-column		MSP-ZEWB 0.2 mg each time	
	Sample, mg/L	Eluent, mg/L	Sample, mg/L	Eluent, mg/L	Sample, mg/L	Eluent, mg/L
Adenine	99.0	ND	99.0	ND	138	2.2
Thymine	98.0	ND	98.0	ND	80	8.2
Adenosine	176	ND	176	ND	176	3.1
Thymidine	163	2.1	163	ND	163	2.1
ATP	83.0	ND	83.0	ND	86	206
AMP	164	ND	164	ND	130	16.4

The sample of each compound in 200 μl of 200 mM sodium acetate at pH 3.6 was applied to each separation media. The compound in 20 μl of the eluent at pH 8.9 in 25 mM Tris–HCl was quantified by absorbance at 260 nm with Thermo-Fisher Nanodrop 1000.

**Note 1**. Selection of ionizable groups and optimization of their molar ratios on AIEMs for concomitant achievement of the maxima of adsorption and elution.

For the purification of ionizable soluble biomacromolecules via the reversal of the types of net charges on the adsorbed biomacromolecules, or the reversal of the types of net charges on the AIEMs, to change electrostatic attractions for the adsorptions to electrostatic repulsions for the elution, a proper AIEM should have three types of net charges over a wide enough pH range, besides high density of small zwitterions as the coats and a large quantity of ampholytic groups (Fig. 1a).

The purification of ionized soluble biomacromolecules with AIEMs relies primarily on electrostatic attractions for adsorptions and electrostatic repulsions for elution. With AIEMs, buffer pH should be limited within 4.0–10.0 for the tolerability/stability of the ion-exchangers and targeted biomacromolecules and facile consecutive processing of the purified biomacromolecules. To concurrently achieve the maxima of adsorption capacity and elution efficacy, ampholytic functionalities on the proper AIEMs can be made of selected ionizable groups, whose combination gives negligible negative charges at pH close to 4.0 while produces negligible positive charges at pH close to 10.0. Such selected groups should exhibit negligible hydrophobicity. Ionizable groups producing negative charges and positive charges are thus sought.

The pH for zero net charge on an AIEM is designated its pIm; the isoelectric point of an ampholytic molecule (protein) or group is denoted its pIt, and the ionization constant (pKa or pKb) of a non-ampholytic ionizable molecule/group is also denoted its pIt. Accordingly, on an ampholytic molecule (protein), there are positive net charges at pH < pIt but negative net charges at pH > pIt. However, there will be negative or zero net charges at a given pH for a non-ampholytic acid group, but positive or zero net charges at a pH for a non-ampholytic basic group; such two types of ionizable groups on proper AIEMs should be carefully selected and engineered with suitable molar ratios to facilitate achieving the maxima for both the adsorption capacity and elution efficacy of the proper AIEMs.

Carboxyl, phosphate and sulfonate are common groups to provide negative charges upon ionization in aqueous solutions. However, phosphate and sulfate have pIts lower than 2.0 and will exhibit significant ionization at pH close to 4.0. Carboxyl has a pIt of about 4.8, and exhibits negligible ionization at pH close to 4.0. Namely, carboxyl is the sole choice on proper AIEMs to produce negative charge upon ionization. Meanwhile, for negligible non-electrostatic interactions, short aliphatic carboxyl is needed. On the other hand, organic amines and imidazole are suitable candidates of groups producing positive charges upon ionization. Similarly, for negligible non-electrostatic interactions, only short aliphatic amines and imidazole are applicable. Moreover, aliphatic secondary and tertiary amines usually show pIts higher than those of primary amines and thus significant ionization even at pH close to 10.0. In fact, such values of pIts are greatly affected by polar substituents and polarity of micro-environments. For example, methylamine has a pIt of about 10.0 while Tris has a pIt of about 8.1, acetic acid has a pIt of about 4.8; however, glycine as an ampholytic compound has one amino and one carboxyl moiety and a pIt of about 6.0, which is much smaller than 7.4 as the mean of pIts of methylamine and acetic acid. Imidazole has a pIt of about 6.0. Short aliphatic amines bearing polar substituents and short aliphatic imidazole are thus two subtypes of suitable groups to produce positive charges on proper AIEMs.

For reproducible SPE, MSPs are preferable. MSPs bearing small zwitterion coats (MSP-ZEW) and short aliphatic carboxyl groups thus served as the starting solid supports; lysine, glycine and N,N-dimethylethylenediamine were the applicable precursors of the required ionizable groups, whose combinations at dynamic ratios gave magnetic AIEMs bearing different pIms (Fig. 1b).

The density of the two types of ionizable groups on AIEMs and their molar ratios determine the numbers of the positive net charges at pH 4.0 and the numbers of the negative net charges at pH 10.0.

In general, the achievement of no less than 80% of the maxima of the numbers of net charges can be acceptable; the molar ratios of those two selected types of ionizable groups should be optimized for the numbers of positive net charges on AIEMs at pH 4.0 close to 80% of their respective maxima while the numbers of negative net charges on those AIEMs at pH 10.0 close to 80% of their respective maxima. For the approximation of the range of molar ratios of those two selected types of ionizable groups on the proper AIEMs, pIts of required short aliphatic carboxyl are thus assumed to be about 4.7 while pIts of required short aliphatic amines are assumed to be about 8.0. Clearly, pIts of ionizable groups producing negative charges solely upon ionization are smaller than pIts of ionizable groups producing positive charges solely upon ionization by no less than 3.0. At pH values smaller than the pIts of required short aliphatic carboxyl by 1.0 unit, its ionization degrees should be no more than 10%. Similarly, at pH values higher than pIts of ionizable groups producing positive charges by 1.0 unit, its ionization degrees should be no more than 10%. However, at pH close to the pIts of the required short aliphatic carboxyl, there will be approximately full ionization of the ionizable groups producing positive charges solely; at pH close to the pIts of the required ionizable groups producing positive charges solely, there will be nearly full ionization of the required short aliphatic carboxyl. The molar ratios of the two selected types of ionizable groups need optimization for the maximal numbers of positive net charges on AIEMs at pH of about 4.0 and the maximal numbers of negative net charges on AIEMs at pH of about 10.0.

The ionization percentage of the required short aliphatic carboxyl should be about 10% at pH close to 3.7; at pH close to 10.0, the ionization of the required short aliphatic amine should be just about 2% and imidazole should exhibit negligible ionization at all. These situations require molar ratios of two suitable types of ionizable groups from 1:3 to 3:1 on proper AIEMs for the maximal numbers of positive net charges on surfaces at pH of about 4.0 and the maximal numbers of negative net charges on surfaces at pH of about 10.0. If the concomitant achievement of the maximal numbers of positive net charges at pH 3.0 and the maximal numbers of negative net charges at pH 11.0 is expected, their molar ratios can vary from 1:12 to 12:1. For general applicability, their molar ratios are set from 1:6 to 6:1 to prepare proper AIEMs bearing dynamic pIms, enabling their promising applications to the purification of most ionizable soluble biomacromolecules.

## 2 Experimental design, materials and methods

### 2.1 Experimental design

For determining surface net charges of the designed AIEMs under different pH and its application for the isolation of ionizable substances, acid red 13 was selected as the anion probe. The quantity of the anion probe disappeared from an adsorption system was assigned to that adsorbed. The minimum adsorption pH giving the adsorption capacity of an AIEM for the anion probe less than 2% of that at pH 3.6 was taken as the pIm. Through the adsorption at varying pH and the elution at pH 8.9, AIEMs showed different pH for no net charges as pIms.

Using two uricases as the protein models to determine the isolation efficiency of AIEMs via the reversal of the type of net charges of uricases or the AIEM to change electrostatic attraction for adsorptions to electrostatic repulsion for elution, the activity yields and homogeneity after purification were compared with the commercial classical ion-exchange media.

Using plasmid of a uricase as the model to determine the efficiency of AIEMs for purification of plasmid via the reversal of the type of net charges on AIEMs to fulfil electrostatic repulsion for efficient elution, qPCR of the purified plasmid was compared; the competitive adsorption of bases, nucleosides and nucleotides on AIEMs were determined.

### 2.2 Materials and methods

MSP bearing carboxyl functionalities and coats of small zwitterion were provided by Chongqing Bolanying Biotechnology Co. Ltd (Chongqing, China; http://www.fbdbio.com). Acid red 13 was purchased from Tokyo Chemical Industry Co. Ltd (Shanghai, China). Toyopearl SP-650C and Toyopearl QAE-550C were provided by Tosoh (Shanghai, China). Dynabeads Myone silane was provided by Thermo-Fisher (Shanghai, China). The reagent kit for magnetic purification of plasmids in cell lysates was provided by Biomiga (Shanghai, China). The plasmids of MGU and BFU were those utilized previously [2], [3]. Other chemicals were analytical reagents.

#### 2.2.1 Preparation of magnetic AIEMs and characterization

To activate carboxyl on MSP-ZEW, dicyclohexanyl carbodiimide (DCC) of 1.0 g plus N-hydroxysuccinamide (NHS) of 1.0 g was added to MSP-ZEW of 1.0 g in 100 ml N,N-dimethylforamide (DMF); the suspension of MSP-ZEW was kept mixing overnight at 25°C before thorough wash of the resulted MSP-ZEW with DMF. For conjugation with 1.0 g of the activated MSP-ZEW at pH 6.0, the use of 30 mM lysine solution (35 ml) plus 30 mM glycine (15 ml) gave MSP-ZEWA, that of 30 mM lysine alone (50 ml) yielded MSP-ZEWB, while that of 15 mM lysine solution (70 ml) plus 15 mM N,N-dimethylethylenediamine (30 ml) produced MSP-ZEWC. The suspensions for each conjugation reaction were kept mixing at 25°C for 2 h, before thorough wash of the resulted media with 20 mM sodium phosphate at pH 7.4.

For the adsorption of proteins or plasmids from a sample prepared in an indicated buffer, AIEMs were mixed with the sample for mild shaking at room temperature in 15 min. After the adsorption, magnetic AIEMs were separated with magnetic separation stands (Promega) in 5.0~10.0 min. For the wash of loosely-bound substances, magnetic AIEMs after adsorption were suspended in the adsorption buffer for mild mixing with pipette in 5.0 min and recovered again. For the elution of the adsorbed substances, magnetic AIEMs were suspended in the elution buffer at an indicated pH for mild shaking in 5.0–15 min and the supernatant were collected after magnetic removal of the corresponding AIEMs.

#### 2.2.2 Uricase expression and activity assay

After the transformation of Escherichia coli BL21 (DE3) with each expression plasmid and the amplification of the transformed cells at 37°C for 4 h, the targeted protein was induced with 0.5 mM isopropyl β-D-thiogalactoside in 16 h at 16°C. After the wash of those cells through centrifugation, lysates were prepared in an indicated adsorption buffer by sonication treatment for 5.0 min at 0°C, which after further centrifugation served as the samples. Uricase activity was determined in 0.20 M sodium borate at pH 9.2 with final 75 μM uric acid to measure the absorbance at 293 nm. Unless otherwise stated, proteins were quantified with Nanodrop 1000.

#### 2.2.3 Purification of MGU plasmid and its qPCR

To purify the plasmid of MGU in cell lysates, the standard protocol with spin silane-column was used. For magnetic separation of the plasmid, the adsorption system was 0.20 ml of 200 mM sodium acetate at pH 3.6 and the elution utilized 50 μl of 25 mM Tris–HCl buffer at pH 8.9. For qPCR, SYBR Green was used with a specific pair of primers giving products of about 150 bp and the standard protocol with Biorad CFX96 was employed; the cycle number thresholds for PCR were derived. The independent assays with the same sample gave cycle number thresholds bearing coefficient of variation below 3%.
